# Metal Ion-Mediated Interfacial Coordination Complexation in Octyl Gallate-Curcumin Emulsions: Enhanced Stability and Curcumin Protection

**DOI:** 10.3390/foods15020265

**Published:** 2026-01-11

**Authors:** Tong Li, Yongting Feng, Rong Huang, Bin Li, Guoqiang Zhang, Hongshan Liang

**Affiliations:** 1 College of Food Science and Technology, Huazhong Agricultural University, Wuhan 430070, China; litong_fse@163.com (T.L.); foodilike@163.com (Y.F.); 17755240901@163.com (R.H.); libinfood@mail.hzau.edu.cn (B.L.); 2 Key Laboratory of Environment Correlative Dietology, Huazhong Agricultural University, Ministry of Education, Wuhan 430070, China; 3 Wuhu Green Food Industry Research Institute Co., Ltd., Wuhu 241000, China

**Keywords:** curcumin, interfacial cross-linking, emulsions, stability

## Abstract

This study developed an efficient interfacial stabilization strategy, using metal ions (Cu^2+^) and octyl gallate (OG) to protect curcumin (Cur) via interfacial coordination. Macroscopic observation, droplet size, and Turbiscan stability index analysis demonstrated that the addition of Cu^2+^ to the OG/Cur emulsion significantly influenced its emulsification efficiency and physical stability, which depended on both the OG concentration and the amount of Cu^2+^ added. Interfacial rheological analysis showed that Cu^2+^ addition significantly enhanced droplet interfacial strength, with distinct effects from different metal ions. FT-IR confirmed the coordination bonds of Cu^2+^ with both Cur (keto/enol) and OG (phenolic hydroxyl). Under appropriate concentrations of OG and Cu^2+^, the retention rate of curcumin in the emulsion was significantly improved under various processing conditions. After 100 min of UV exposure, the OG/Cur/Cu^2+^ system increased curcumin retention by 49.64% compared to Cu^2+^-free systems. The study presents a metal-phenolic coordination-based strategy for constructing stable functional emulsions with high curcumin protection.

## 1. Introduction

Curcumin is a natural bioactive compound with significant potential in functional foods due to its health benefits [1,2,3]. However, its application is hindered by poor water solubility and low stability against environmental stressors such as light, heat, and pH variations [4]. Encapsulation techniques, including emulsions, liposomes, and nanoparticles, offer effective protection for bioactive compounds against environmental degradation. Emulsion encapsulation is an effective strategy to enhance the solubility and stability of lipophilic compounds like curcumin [5,6]. Conventionally, emulsion stability relies on amphiphilic emulsifiers (such as surfactants, proteins, or polysaccharides), which reduce interfacial tension and provide electrostatic repulsion, thereby preventing droplet coalescence [7,8]. Nevertheless, such interfaces are often formed through dynamic adsorption and may lack sufficient mechanical strength. This typically results in poor long-term stability of the emulsion and an inability to prevent the degradation of encapsulated bioactive substances under complex processing conditions [9]. Therefore, novel approaches are required to engineer robust interfacial structures.

To address this, polyphenol-metal ion coordination has emerged as a promising strategy to construct robust interfacial films in food delivery systems [10,11]. This approach is grounded in the coordination chemistry equilibrium between metal ions and polyphenol ligands, which can lead to the in situ formation of viscoelastic, cross-linked networks whose density and strength are governed by ion concentration, pH, and ligand affinity [12]. Notably, the β-diketone moiety within curcumin exhibits strong chelating capacity, enabling interactions with diverse metal ions to form complexes exhibiting superior stability [13]. This suggests the potential dual role of curcumin as both the core bioactive compound and an active component of the interfacial architecture. Furthermore, as amphiphilic polyphenol derivatives, gallates (such as octyl gallate) can not only function as emulsifiers on their own but also coordinate with metal ions to form a more stable interface. Additionally, gallates possess antioxidant and UV resistance capabilities [14].

Based on the above theory, we hypothesize that the formation of coordination complexes between metal ions, octyl gallate, and curcumin will concurrently enhance the stability of both the oil-water interface and curcumin. Therefore, this study introduces an interfacial coordination strategy using octyl gallate and metal ions to stabilize curcumin-loaded emulsions. We systematically investigated the effects of emulsifier concentration and metal ion concentration/type on emulsion properties and stabilization mechanisms.

## 2. Materials and Methods

### 2.1. Materials

Curcumin (biological purity), octyl gallate (HPLC ≥ 98%), and MOPS (ultrapure, 99.5%) were purchased from Shanghai Yuanye Bio-Technology Co., Ltd. (Shanghai, China). Corn germ oil was obtained from Yihai Kerry Arawana Oils & Grains Co., Ltd. (Shanghai, China). Copper chloride, ferrous sulfate, zinc sulfate heptahydrate, anhydrous magnesium chloride, and anhydrous calcium chloride, all of analytical grade, were acquired from Sinopharm Chemical Reagent Co., Ltd. (Shanghai, China). Ultrapure water (18 MΩ⋅cm) was used throughout the experiments.

### 2.2. Preparation and Characterization of Curcumin Emulsions

#### 2.2.1. Preparation of Gallate-Stabilized Curcumin Emulsions

Curcumin (1 mg/mL) and octyl gallate were dissolved in corn oil to form the oil phase. Based on preliminary experiments, the oil phase containing 2 wt% octyl gallate was added dropwise into the aqueous phase at an oil-to-water ratio of 1:9 (Figures S1 and S2). An ultrasonic disruptor (Sonics & Materials, Inc., Newtown, CT, USA) was used at 80 W with a working cycle of 1 min on and 5 s off for a total duration of 3 min.

#### 2.2.2. Preparation of Octyl Gallate- and Metal Ion-Stabilized Curcumin Emulsions

Based on Section 2.2.1, the mass fraction of octyl gallate in the oil phase was adjusted (0.1 wt%, 0.2 wt%, 0.3 wt%, 0.4 wt%). The oil-to-water phase ratio was 1:9 (*v*/*v*). Subsequently, specific volumes (80 μL, 100 μL, 150 μL) of 24 mM metal ions (Ca^2+^, Mg^2+^, Zn^2+^, Cu^2+^, Fe^2+^) were added under stirring, followed by continued sonication for another 3 min to prepare the upgraded emulsions.

### 2.3. Visual Assessment of Emulsions

Curcumin emulsion samples were stored at room temperature, and the visual appearance changes in the emulsion were monitored and photographed during storage to track any phase separation behavior [15].

### 2.4. Droplet Size and Zeta Potential of Emulsions

The emulsion was diluted 100-fold with MOPS buffer (0.01 M). The droplet size and zeta potential of the emulsions were determined via dynamic light scattering (DLS) using a nano-laser particle size analyzer (NanoZS, Malvern Instruments, Malvern, UK) [16].

### 2.5. Backscattering and Turbiscan Stability Index of Emulsions

Stability analysis of the emulsions was performed using a multiple light scattering instrument (TOWER, Formulaction SA, Toulouse, France). For the overall Turbiscan stability index (TSI) stability assessment of the curcumin emulsions, samples (20 mL) were loaded into cylindrical glass cells and analyzed at 25 °C for 3 h, with scans performed at 10 min intervals. Additionally, samples were continuously monitored over one week to obtain their backscattered light profiles [17].

### 2.6. Interfacial Rheology Property Measurement

The adsorption behavior of phenolic hydroxyl-metal ion-keto/enol complexes at the oil-water interface was investigated, with Medium-chain triglyceride (MCT) oil as the oil phase, using an interfacial rheometer (Tracker Teclis, Teclis Instruments, Lyon, France), according to the previous paper [18].

Dynamic Interfacial Pressure Measurement: 25 mL aqueous phase was added to the glass cell. The microsyringe (500 μL) filled with Medium-chain triglyceride (MCT) oil was inserted. The droplet (10 μL) was formed at the needle tip via a motor-driven system. Interfacial tension (γ) was monitored as a function of adsorption time (t) for 10,800 s at 25 °C. The rearrangement processes of LFblg/SFblg-Ca^2+^ complexes from the aqueous phase to the oil-water interface were further investigated using the following equation. The interfacial adsorption kinetics were further studied by using the following formula:

(1) $$\;\pi \;={\;\gamma }_{0}-\;\gamma$$

(2) $$\pi \;=\;K_{\mathrm{diff}}\;\times \;t_{1/2}$$

Interfacial Dilational Rheology: The syringe was oscillated sinusoidally to induce interfacial deformation. Five effective cycles and five blank cycles were run within the linear viscoelastic regime. Prior to frequency sweep measurements, amplitude sweep tests were conducted at a constant frequency (0.1 Hz) to determine the linear viscoelastic region of the interfacial layer. The test conditions were: 10% of deformation amplitude within the linear regime, 0.1 Hz of oscillation frequency, duration 7200 s, and temperature 25 °C. The interface modulus E is calculated by the following formula:

(3) $$E=\frac{d\pi \;}{dlnA}$$

where γ_0_ is the interfacial tension of pure solvents, γ is the interfacial tension of the system, π is the interfacial pressure, K_diff_ is the rate of diffusion, and A is the drop’s surface area.

### 2.7. UV–Visible Spectroscopy

The binding ratios of curcumin–metal and octyl gallate–metal were investigated by UV–Visible spectrophotometry. Curcumin was dissolved in ethanol (1 mg/mL) and mixed with the buffer solution at a 1:9 ratio under stirring. Metal ion solutions of varying concentrations were then added dropwise to prepare curcumin–metal ion complexes. Similarly, octyl gallate was dissolved in ethanol (0.3 wt%) and mixed with buffer (1:9 ratio), followed by dropwise addition of different metal ion concentrations to obtain octyl gallate–metal ion complexes. Absorbance was measured at their respective maximum absorption wavelengths using a UV-Vis spectrophotometer (UV-1800, Shimadzu Corporation, Kyoto, Japan).

### 2.8. Fourier Transform Infrared Spectroscopy (FTIR)

The interactions among octyl gallate, curcumin, and Cu^2+^ were analyzed using FTIR (Jasco 4100, JASCO Corporation, Oklahoma City, OK, USA). The analytical protocol was adapted from Wu et al. [19]. The curcumin emulsion prepared with 0.3 wt% octyl gallate as the emulsifier, an oil-water ratio of 1:9, and an addition amount of 100 μL of Cu^2+^ was used as the sample. Among them, corn oil is replaced with anhydrous ethanol. The dried samples were mixed with KBr at a mass ratio of 1:100 and ground thoroughly. FTIR spectra were recorded from 4000–400 cm^−1^ using a blank KBr pellet as background.

### 2.9. The Stability of the Emulsion and Curcumin Under Different Treatment Conditions

#### 2.9.1. Storage Stability

The samples were stored in the dark at 4 °C, 25 °C, 37 °C, and 50 °C, respectively. The particle size of the emulsion and the retention rate of curcumin in the samples were measured regularly. The formula for testing the retention rate is as follows:

(4) $$\mathrm{Retention}\;\mathrm{rate}\;(\%)\;=\frac{C_{t}}{C_{0}}\times 100$$

where retention rate (%) refers to the retention rate of curcumin, C_t_ is the total concentration of curcumin measured after t days of storage, and C_0_ is the initial total concentration of curcumin in the sample.

#### 2.9.2. Thermal Stability

The samples were heated in a water bath at 60 °C, 70 °C, 80 °C, and 90 °C for 30 min, respectively. After removal, the particle size of the emulsions was measured, and the retention rate of curcumin was determined.

#### 2.9.3. pH Stability

The pH of the curcumin emulsion was adjusted to 5.5, 6.5 and 7 using 1 mol/L HCl or NaOH, respectively. The macroscopic changes in the emulsion were observed. The emulsion was stored in the dark at room temperature, and its particle size and the retention rate of curcumin were determined.

#### 2.9.4. Photochemical Stability

The samples were exposed to ultraviolet light for 100 min. Samples were taken every 20 min to determine the retention rate of curcumin.

### 2.10. Statistical Analysis

All experimental data in this study are expressed as mean ± standard deviation (SD) from three independent preparations of each sample. Statistical analysis was performed via SPSS 26.0 software using analysis of variance (ANOVA). The significance level was set at *p* ≤ 0.05. Graphical plotting was conducted using Origin 2022.

## 3. Results

### 3.1. Factors Influencing the Performance of Metal Ion-Coordinated Emulsions

#### 3.1.1. Effect of Octyl Gallate Concentration

**Figure 1:**
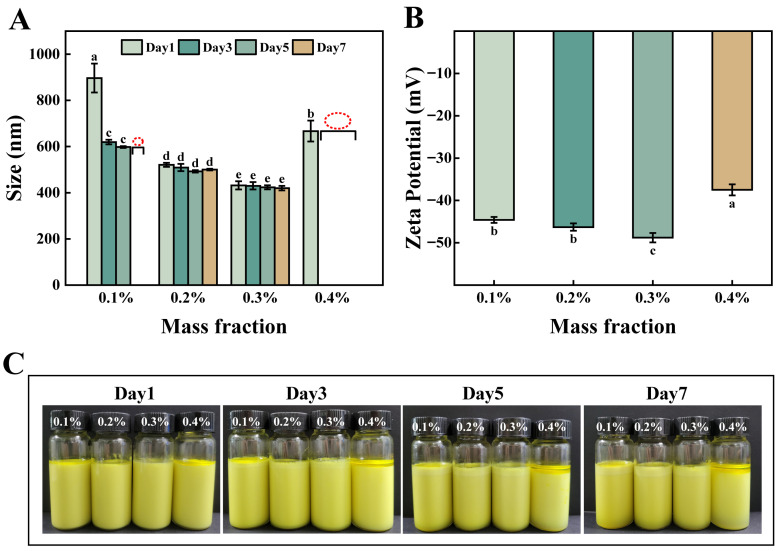
Particle size (**A**), Zeta-potential (**B**), visual appearance (**C**) of emulsions with different octyl gallate addition amounts. Different lowercase letters (a–e) indicate statistically significant differences (*p* ≤ 0.05).

Preliminary experiments determined the required concentration of octyl gallate (OG) as the sole emulsifier for emulsion formation (Figures S1 and S2). Following the introduction of Cu^2+^ (100 μL), the impact of OG mass fraction (0.1–0.4 wt%) on the emulsion system was re-evaluated. As shown in Figure 1A, emulsions containing 0.1 wt% OG exhibited a large initial droplet size (878 nm on Day 1). By Day 7, significant phase separation occurred, characterized by oiling-off at the top and water separation at the bottom (Figure 1C), indicating complete loss of physical stability. The appearance of an oil film at the top indicated severe aggregative instability, while the separation of the aqueous phase at the bottom is a direct result of sedimentation instability. The decrease in emulsion droplet size over time could be attributed to the aggregation and eventual phase separation of larger droplets, resulting in a decrease in the measured average droplet size [20]. This instability suggests insufficient emulsifier concentration, leading to incomplete interfacial coverage and a loosely packed interface. Consequently, the interfacial tension failed to reach its minimum value, compromising physical stability [21]. Increasing the OG concentration to 0.2 wt% significantly reduced droplet size, demonstrating effective emulsifier adsorption at the oil-water interface. The concentration (0.2 wt%) facilitated rapid adsorption onto lipid droplet surfaces, thereby preventing droplet aggregation [22]. A further increase to 0.3 wt% resulted in a marginal size reduction and minimal size change over time (Figure 1A). Additionally, electrokinetic data shows that when the emulsifier was at 0.3 wt%, the absolute value of the zeta potential was the highest, indicating the strongest repulsive force between particles (Figure 1B), which can inhibit particle aggregation and lead to a smaller particle size [23]. Macroscopic images further confirmed that the sample at 0.3 wt% was more stable than the condition at 0.2 wt%. However, raising the OG concentration to 0.4 wt% drastically increased droplet size (653 nm) and induced aggregative instability by Day 3, evidenced by substantial free oil at the surface. This phenomenon might be attributed to exceeding the optimal emulsifier concentration (approximately 0.3 wt% under these conditions). The excess OG molecules can adsorb onto the surfaces of two or more droplets, inducing cross-linking between them and thereby compromising the stability of the emulsion [24].

**Figure 2:**
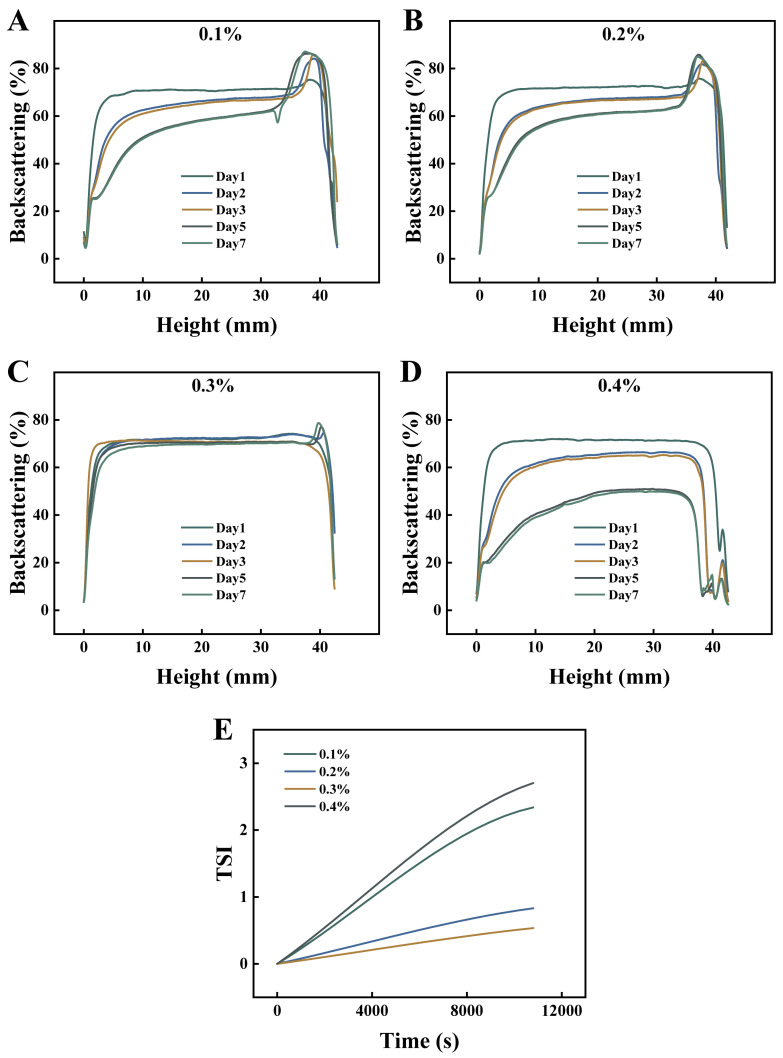
Backscattering (**A**–**D**) and Turbiscan stability index (**E**) of emulsions at different octyl gallate concentrations.

Figure 2E shows the Turbiscan Stability Index (TSI) over time. The 0.3 wt% octyl gallate emulsion had the lowest TSI values overall. The TSI curve rose slowly during storage, showing the curcumin emulsion remained stable. All samples were monitored for seven days using multiple light scattering (Figure 2A–D). Emulsions with 0.1 wt% and 0.2 wt% octyl gallate showed matching backscattering (BS) patterns, which revealed decreasing BS at the bottom and middle, and increasing BS at the top. This signature profile demonstrates droplet creaming, with concomitant water separation at the bottom and oiling-off at the top. Concurrent depression of BS curves in the middle and bottom layers signified droplet aggregation and reduced droplet density in these regions. Excessive droplet aggregation results in complete phase separation [25]. In contrast, the 0.3 wt% system displayed tightly superimposed BS curves across all heights, confirming exceptional stability. Conversely, the 0.4 wt% emulsion exhibited pronounced BS depression throughout the column with increasing magnitude, diagnostic of comprehensive emulsion breakdown [26]. These analytical findings align precisely with macroscopic observations.

#### 3.1.2. Effect of Cu^2+^ Addition Amount

**Figure 3:**
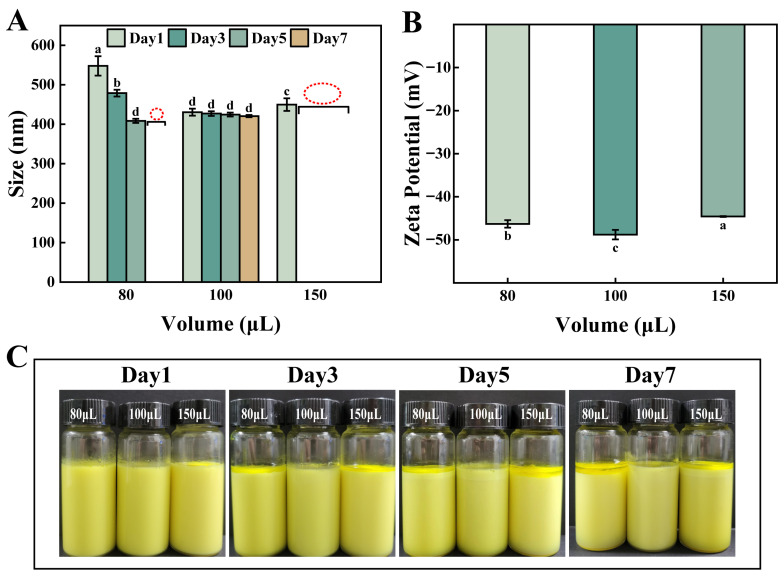
Particle size (**A**), Zeta potential (**B**), and macroscopic images (**C**) of emulsions with different Cu^2+^ addition amounts. Different lowercase letters (a–d) indicate statistically significant differences (*p* ≤ 0.05).

Figure 3A shows the variation in emulsion particle size with storage time under different Cu^2+^ addition amounts (80 μL, 100 μL, 150 μL). When Cu^2+^ addition was 80 μL, the particle size was significantly larger than that of other groups on Day 1, and subsequently underwent substantial changes over time. When the addition was 100 μL, the particle size remained relatively small over 7 days, from 431 nm on Day 1 to 421 nm on Day 7. When the Cu^2+^ addition increased to 150 μL, the particle size could not be reliably measured by the third day due to the disruption of the emulsion. Figure 3B shows that at 100 μL of Cu^2+^, the negative charge was highest at −48.8 mV. Additionally, macroscopic images (Figure 3C) show that the emulsion at 100 μL of Cu^2+^ remained stable and uniformly dispersed over 7 days. The emulsion with 80 μL Cu^2+^ showed obvious separation by Day 7. At 150 μL of Cu^2+^, oil droplets appeared on the surface, indicating that high metal ion concentration led to instability [24].

**Figure 4:**
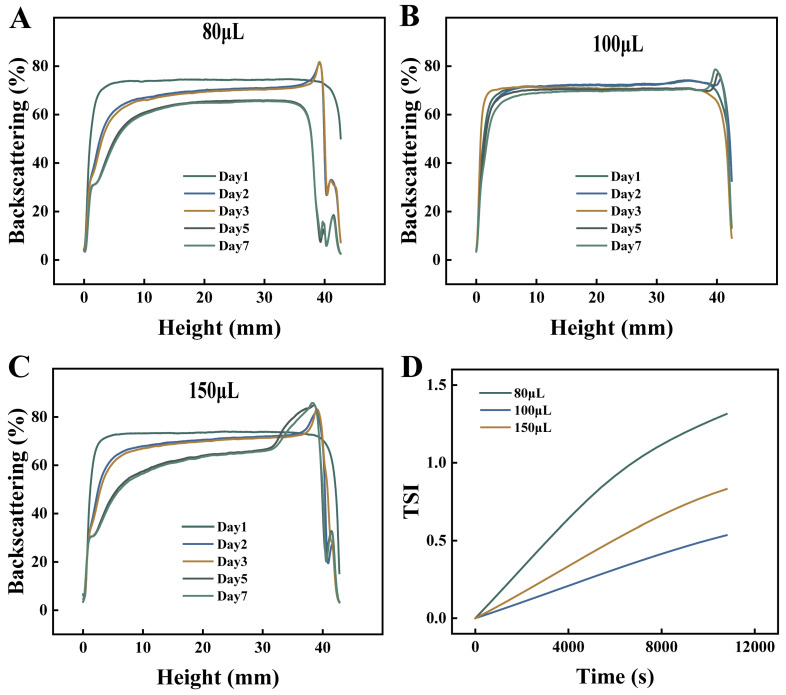
Backscattering (**A**–**C**) and Turbiscan stability index (**D**) of emulsions with different Cu^2+^ addition amounts.

The Turbiscan stability index (TSI) quantitatively characterizes emulsion stability, where lower values and slower temporal increases indicate superior stability [27]. As shown in Figure 4D, the system containing 100 μL Cu^2+^ exhibited the most gradual TSI increase, demonstrating optimal kinetic stability. In contrast, systems with either insufficient (80 μL) or excessive (150 μL) Cu^2+^ showed rapid TSI elevation, indicating compromised stability that correlated well with macroscopic observations and particle size measurements.

Backscattering intensity profiles reflect the spatial homogeneity of emulsion droplet distribution, with flatter curves indicating more uniform dispersion. For the system with 80 μL Cu^2+^ (Figure 4A), slight fluctuations in backscattering curves were observed during Days 1–3, showing decreased intensity at the bottom/middle layers and increased intensity at the top. More pronounced curve variations emerged during Days 5–7, indicating initial droplet coalescence and phase separation [28]. It aligned with visible stratification observed macroscopically on Day 7 (Figure 3C). These findings demonstrate that although 80 μL of Cu^2+^ provided partial interfacial reinforcement, the quantity was insufficient for complete oil-water interface coverage. This lack of full coverage thus led to eventual instability. In contrast, the 100 μL of Cu^2+^ system (Figure 4B) maintained highly overlapping and stable backscattering curves throughout storage, demonstrating excellent long-term homogeneity. When combined with the nearly constant particle size data, these results can likely be attributed to the enhanced coordination effect at elevated Cu^2+^ concentrations, which stabilized the oil-water interface and thereby effectively prevented droplet aggregation and phase separation. However, when the Cu^2+^ concentration was further increased to 150 μL (Figure 4C), the emulsion exhibited a sharp rise in backscattering intensity at the top layer, accompanied by a significant decrease at the bottom after Day 3, which demonstrates characteristic phase separation behavior. This phenomenon showed strong correlation with both macroscopic emulsion breakdown (Figure 3C) and invalid particle size measurements (Figure 3A). These collective observations suggest that excessive Cu^2+^ concentrations may induce irreversible droplet coalescence and phase separation in the emulsion system.

#### 3.1.3. Effect of Metal Ion Species

**Figure 5:**
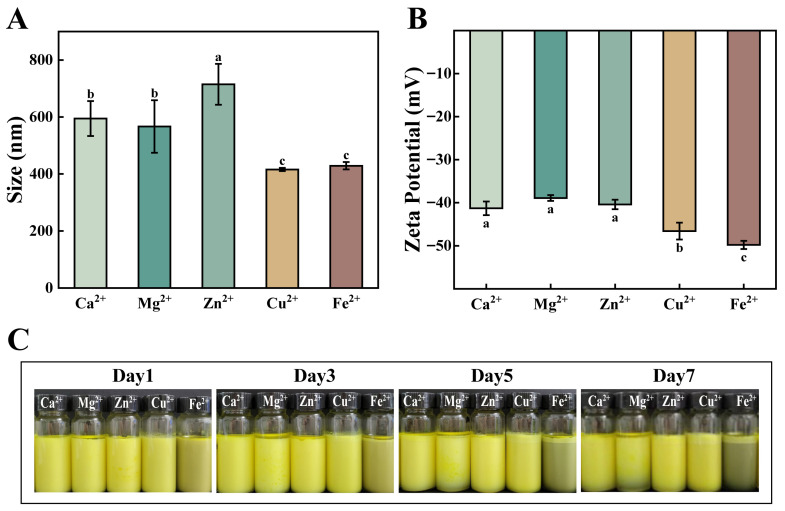
Particle size (**A**), zeta potential (**B**) and macroscopic images (**C**) of emulsions with different types of metal ions. Different lowercase letters (a–c) indicate statistically significant differences (*p* ≤ 0.05).

The 1,3-dicarbonyl group of curcumin can easily chelate metal ions such as Cu^2+^, Fe^2+^, and Zn^2+^, forming complexes, which helps prevent curcumin from degrading due to environmental influences. Different metal ions have varying protective effects on curcumin, so the effects of different metal ions on curcumin emulsions were investigated. According to Figure 5C, emulsions prepared with Cu^2+^, Zn^2+^, and Ca^2+^ showed good physical stability during the assessment period, while Mg^2+^-prepared emulsions showed oil separation on the first day. It has been suggested that Mg^2+^ may cause oil droplets to aggregate and shield electrostatic repulsion through hydrogen bonding, leading to chain collapse and reduced spatial stability [29]. Fe^2+^-prepared emulsions showed oil separation on the third day. Particle size results (Figure 5A) showed that Cu^2+^-prepared emulsions had the smallest particle size (416 nm) compared to other metal ion emulsions, and they had the highest absolute zeta potential (Figure 5B), indicating that Cu^2+^ inhibits flocculation and aggregation of droplets during emulsification, leading to smaller particle size [30]. Overall, Cu^2+^-prepared emulsions showed better overall stability.

### 3.2. Interfacial Coordination Analysis

#### 3.2.1. Analysis of Cu^2+^-Mediated Interfacial Rheology

Interfacial rheology was used to analyze changes in interfacial tension and modulus, in order to investigate the effect of Cu^2+^ concentration on the system’s interfacial properties.

**Figure 6:**
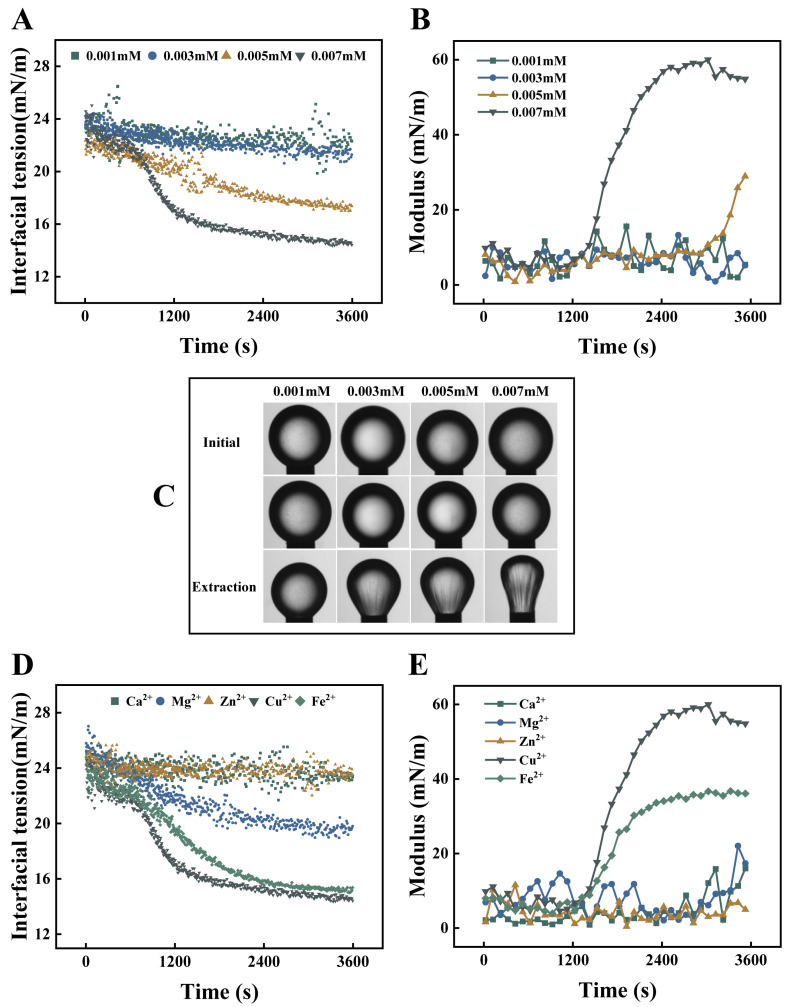
Interfacial tension (**A**), state diagram of droplets during the adsorption process (**B**), dilational viscoelastic modulus (**C**) at different concentrations of Cu^2+^; interfacial tension (**D**), dilational viscoelastic modulus (**E**) for different types of metal ions.

Interfacial tension reflects the energy state at oil-water interfaces, where lower values indicate greater stability. This property depends directly on emulsion composition [31]. As shown in Figure 6A, at low Cu^2+^ concentrations (0.001–0.003 mM), interfacial tension (IT) showed minimal reduction, stabilizing at 22 mN/m, which indicated weak coordination between Cu^2+^ and OG/Cur. Higher concentrations (0.005–0.007 mM) induced rapid IT reduction, with faster kinetics at increased levels. It is attributed to bidentate chelation between Cu^2+^ and phenolic hydroxyl groups as well as the ketone/enol structures that markedly lowered interfacial polarity [32,33]. Similarly, the diffusion coefficients (K_diff_) obtained by fitting π with t^1/2^ also showed the same trend. As shown in Table 1, the apparent diffusion coefficients (K_diff_) increased with higher Cu^2+^ concentrations. This phenomenon suggested that the adsorption process was accelerated by interfacial coordination reactions. The presence of Cu^2+^ ions at the interface provided immediate coordination sites for OG and curcumin molecules [34]. However, Figure S3 revealed abnormal interfacial behavior at 0.008 mM Cu^2+^. Between 1500–1800 s, the interfacial tension (IT) first surged beyond 500 mN/m and then dropped sharply below −100 mN/m. This instability resulted from the formation of an interfacial film with high elasticity but low viscosity, causing droplet deformation that no longer satisfied the Laplace equation requirements of the interfacial rheometer [35]. The low-viscosity interfacial film formed by excessive Cu^2+^ concentrations exhibited poor recovery after deformation and eventually ruptured, which was consistent with the observed macroscopic changes and particle size variations.

The droplet morphological changes during initial, equilibrium, and extraction phases revealed the mechanical properties and adsorption stability of interfacial films (Figure 6C). At initial and equilibrium states, droplets maintained perfect spherical shapes with smooth surfaces across all Cu^2+^ concentrations. During extraction, increasing Cu^2+^ concentrations induced more pronounced droplet wrinkling, which demonstrates enhanced interfacial network rigidity through metal-phenolic coordination.

The interfacial modulus reflects the viscoelastic properties of the interfacial film, where higher values indicate greater film toughness and deformation resistance, thereby enhancing emulsion stability. The interfacial modulus analysis provides dynamic insights into how Cu^2+^ enhances the deformation resistance of emulsion interfacial films. As shown in Figure 6B, the temporal evolution of interfacial modulus displayed a distinct concentration threshold effect. At low-to-medium concentrations (≤0.005 mM), the modulus remained consistently low level (<30 mN/m). However, when the concentration reached 0.007 mM, a sharp modulus increase occurred after 1200 s, eventually stabilizing at 50–60 mN/m. This behavior confirms that Cu^2+^-crosslinked interfacial films at this optimal concentration possess superior elasticity and shear resistance. These rheological observations strongly support the interfacial tension and adsorption results.

#### 3.2.2. Interfacial Rheology Mediated by Other Metal Ions

The changes in interfacial properties induced by other metal ions were further investigated. As shown in Figure 6D, interfacial tension exhibited distinct time-dependent patterns across different metal ion systems, with all formulations showing decreasing values. This reduction suggests stable interfacial film formation in O/W emulsions. The stabilizing efficacy of metal ions followed this order: Cu^2+^ > Fe^2+^ > Mg^2+^ > Ca^2+^ ≈ Zn^2+^. Notably, systems containing Cu^2+^ and Fe^2+^ demonstrated the most rapid tension reduction, indicating superior interfacial energy minimization. This phenomenon is attributed to the strong coordination capability of these ions, which facilitates the formation of coordination compounds with OG and Cur [36,37]. Such molecular interactions enhance cross-linking density, thereby stabilizing the oil-water interface. Similarly, the diffusion coefficients (K_diff_) obtained by fitting π with t^1/2^ also showed the same trend. As shown in Table 2, the larger diffusion coefficients (K_diff_) for the Cu^2+^ and Fe^2+^ systems demonstrated that their superior coordination capability accelerated the adsorption of the complexes at the oil-water interface [20].

As shown in Figure 6E, the interfacial modulus exhibited distinct time-dependent trends depending on the metal ion species. Previous studies have demonstrated that different metal ions form chelates with tannic acid, resulting in varying Young’s modulus values [38]. The addition of Cu^2+^ and Fe^2+^ induced a sharp increase in interfacial modulus, suggesting rapid interfacial adsorption and coverage of droplet surfaces, effectively preventing droplet aggregation. This observation aligns with the smaller droplet sizes observed in Cu^2+^-stabilized emulsions [39]. Furthermore, the high interfacial adsorption capacity facilitated the formation of robust physical and electrostatic barriers at the interface, significantly enhancing interfacial strength and resistance [23]. In contrast, systems containing other metal ions exhibited only gradual modulus changes, indicating weaker interfacial film formation. These findings were consistent with the interfacial tension results, confirming that Cu^2+^ provided superior stabilization through enhanced interfacial interactions.

#### 3.2.3. FTIR Spectra Analysis

**Figure 7:**
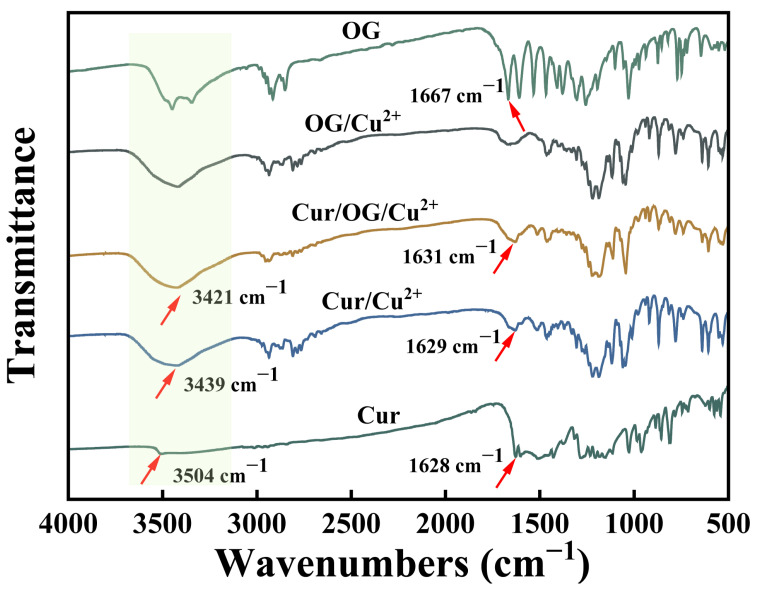
FTIR spectral graph of the interactions among emulsion components.

The interfacial binding mode in the system was analyzed by FTIR spectroscopy. The FTIR spectra of pure compounds (Cur, OG) and their complexes (Cur/Cu^2+^, OG/Cu^2+^, Cur/OG/Cu^2+^) are presented in Figure 7. For curcumin, the ketone group (C=O) stretching vibration at 1628 cm^−1^ [5] showed a blue shift and significant peak broadening in Cur/Cu^2+^ and Cur/OG/Cu^2+^ complexes, likely due to increased bond order induced by Cu^2+^ coordination [40]. Additionally, the characteristic -OH stretching vibration peak at 3504 cm^−1^ [41] exhibited both a red shift and peak broadening in Cur/Cu^2+^ and Cur/OG/Cu^2+^ complexes, which might be due to the involvement of the hydroxyl group from the enol structure of curcumin in coordination [42]. These changes collectively demonstrate that curcumin successfully coordinated with Cu^2+^ in its keto/enol form.

The FTIR spectrum of OG exhibited a characteristic doublet peak in the range of 3450–3300 cm^−1^, attributed to the phenolic hydroxyl groups [43]. In both the OG/Cu^2+^ and Cur/OG/Cu^2+^ complexes, these peaks merged into a single broad peak, likely due to the formation of coordination bonds between the phenolic hydroxyl groups and Cu^2+^ [44]. OG also showed a C=O absorption peak at 1667 cm^−1^, assigned to the ester group [45], which underwent changes in the OG/Cu^2+^ and Cur/OG/Cu^2+^ complexes. Additionally, the multiple peaks observed in the region of 1560–1620 cm^−1^, attributed to C=C resonance in the benzene ring [46], were reduced to a single peak in the complexes (OG/Cu^2+^ and Cur/OG/Cu^2+^). These changes are likely caused by the electron-withdrawing effect of the coordination bonds, further confirming the successful formation of coordination complexes between OG and Cu^2+^.

The FTIR analysis revealed that Cu^2+^ formed stable complexes through coordination with phenolic hydroxyl groups (OG-dominated) and keto/enol structures (Cur-dominated). The vibrational responses of functional groups (peak shifts, intensity changes) provided direct molecular evidence for the coordination mechanisms.

#### 3.2.4. Analysis of Binding Ratios

**Figure 8:**
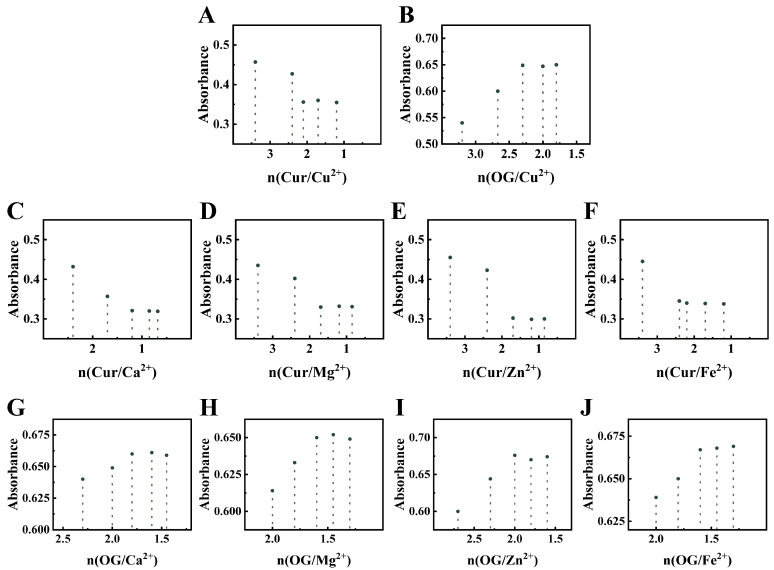
Absorbance of curcumin (Cur) and metal ions at different ratios, Cu^2+^ (**A**), Ca^2+^ (**C**), Mg^2+^ (**D**), Zn^2+^ (**E**), and Fe^2+^ (**F**), respectively. Absorbance of octyl gallate (OG) and metal ions at different ratios, Cu^2+^ (**B**), Ca^2+^ (**G**), Mg^2+^ (**H**), Zn^2+^ (**I**), and Fe^2+^ (**J**), respectively.

Metal-phenolic coordination can occur even under conditions below the ligand’s pKa, because the strong electron affinity of the metal ion can attract the electron density of the hydroxyl group [47]. This facilitates the deprotonation of the hydroxyl group, enabling its direct conversion into a phenolate anion for subsequent binding to the metal ion. The dissociation constants (K_d_) for the gallic acid-Cu^2+^ and curcumin-Cu^2+^ complexes are as low as approximately 2 × 10^−14^ M and 2.9 × 10^−15^ M, respectively. These extremely low Kd values indicate that the formed coordination bonds are exceptionally strong, and the complexes remain virtually undissociated at equilibrium [48,49]. The binding characteristics of curcumin (Cur) and octyl gallate (OG) with metal ions were investigated by monitoring absorbance changes. The ligand-to-metal molar ratio (n) was varied, and the inflection point observed in the absorbance curve corresponds to the saturation state of the ligand-metal complexes. The value of n at this inflection point approximates the binding stoichiometry (Figure 8). For the Cur-metal ions systems, the binding ratio between Cur and Cu^2+^/Mg^2+^/Zn^2+^/Fe^2+^ was approximately 2:1, whereas that for Cur and Ca^2+^ was approximately 1:1 [50]. For the OG-metal ions systems, the binding ratio of OG to Cu^2+^, Ca^2+^, and Zn^2+^ was approximately 2:1. In contrast, the ratio for OG with Mg^2+^ and Fe^2+^ was approximately 1.5:1; this discrepancy may be attributed to stepwise coordination [51].

### 3.3. The Stability of the Emulsion and Curcumin Under Different Treatment Conditions

#### 3.3.1. Storage Stability

**Figure 9:**
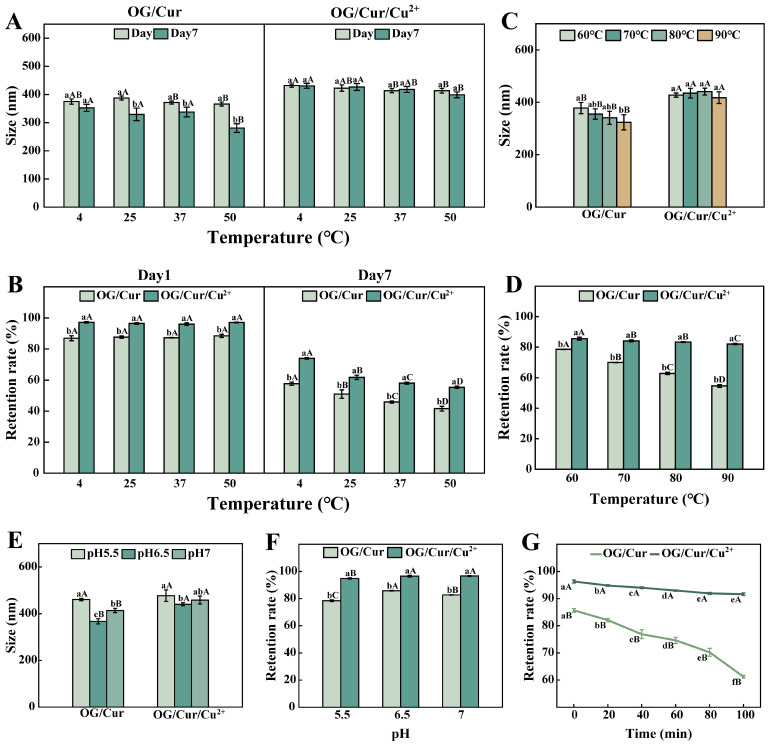
Particle size and retention rate of samples (OG/Cur, OG/Cur/Cu^2+^) under different storage conditions, including storage temperature (**A**,**B**), heating temperature (**C**,**D**), pH conditions (**E**,**F**), and light exposure duration (**G**). Lowercase letters indicate intra-group differences, and uppercase letters indicate inter-group differences (*p* ≤ 0.05).

Figure 9A compares the particle size differences between the OG/Cur system (left) and the OG/Cur/Cu^2+^ system (right) under various storage temperatures (4 °C, 25 °C, 37 °C, and 50 °C) and time points (Day 1, Day 7). For the OG/Cur system, fluctuations in the particle size of the samples were observed during a one-week period across a range of four storage temperatures. In contrast, the OG/Cur/Cu^2+^ system demonstrated excellent stability at all storage temperatures, with no significant differences in particle size between Day 1 and Day 7. Notably, aggregates assembled from tannic acid and nobiletin also maintained stable particle sizes after 72 h of storage at 4 °C and 10 °C, which was attributed to enhanced hydrogen bonding at low temperatures [52].

Furthermore, curcumin retention rates were measured for both systems (Figure 9B). Data from Day 1 and Day 7 consistently showed higher retention rates in the OG/Cur/Cu^2+^ system compared to the OG/Cur system, confirming that Cu^2+^ reinforcement improved interfacial film properties and enhanced curcumin protection. However, all samples exhibited significant declines in curcumin retention over time, which was consistent with the finding reported by Sun et al. [53]. Among the four storage temperatures, the OG/Cur system achieved its best retention performance at 4 °C, with rates of 86.90% (Day 1) and 57.70% (Day 7). Nevertheless, the OG/Cur/Cu^2+^ system maintained stable retention (>92%) across all temperatures on Day 1, but showed noticeable decreases by Day 7, though still significantly higher than the OG/Cur system. Specifically, at the storage temperature of 4 °C, the retention rate of the OG/Cur/Cu^2+^ system decreased from 97.17% (Day 1) to 73.97% (Day 7), a 23.88% reduction. Overall, the 4 °C proved to be the most favorable storage condition for maintaining the stability of the OG/Cur/Cu^2+^ system.

#### 3.3.2. Thermal Stability

Figure 9C compares the particle size differences between the OG/Cur and OG/Cur/Cu^2+^ systems under various heating temperatures (60 °C, 70 °C, 80 °C, and 90 °C). In the OG/Cur system, particle size gradually decreased with increasing temperature. In contrast, the OG/Cur/Cu^2+^ system maintained much more stable particle sizes (418–441 nm) across all heating conditions. This stability arises from the bidentate coordination bonds formed between Cu^2+^ and OG’s ortho-phenolic hydroxyl groups, as well as Cur’s keto/enol structures, which create a more elastic and heat-resistant interfacial structure (FTIR). Similarly, Sun et al. observed minimal particle size changes in OL-EGCG/PC emulsions after 30 min of heating at 85 °C, demonstrating improved thermal stability [53].

Curcumin retention rates fluctuated with increasing temperature (Figure 9D). With the increase in heating temperature, the OG/Cur system exhibited a significant decline in retention, dropping to just 54.63% at 90 °C, which indicates poor heat tolerance. Conversely, the OG/Cur/Cu^2+^ system maintained stable retention (>80%) across 60–90 °C, further confirming its superior protective effect on curcumin under thermal stress.

#### 3.3.3. The pH Stability

Studies have shown that approximately 90% of curcumin degrades within half an hour under pH > 7.2 conditions, generating byproducts [36]. Figure 9E illustrates the differential regulation of pH (5.5–7) on the particle size of OG/Cur and OG/Cur/Cu^2+^ systems. For the OG/Cur system, particle size increased when pH decreased from 6.5 to 5.5 or increased to 7. The OG/Cur/Cu^2+^ system exhibited particle size increases upon pH variation compared with the OG/Cur system, attributable to pH-induced alterations in the ternary coordination complex formation [54]. Figure 9F displays curcumin retention rates under different pH conditions. The OG/Cur/Cu^2+^ system consistently exhibited higher retention than OG/Cur across all pH levels, which suggests a superior protective effect of the OG/Cur/Cu^2+^ system on curcumin against pH variations.

#### 3.3.4. Light Stability

It is well known that curcumin degrades under light exposure. Both systems were exposed to UV light, and the curcumin retention rates are shown in Figure 9G. Overall, the OG/Cur/Cu^2+^ system exhibited higher curcumin retention than the OG/Cur system. As irradiation time increased, retention decreased in both systems. In the OG/Cur system, retention dropped from 82.07% (20 min) to 61.23% (100 min), with a 25.39% decrease. In contrast, the OG/Cur/Cu^2+^ system declined only slightly from 96.3% to 91.63% (a 4.67% reduction). This reduction was 80.90% smaller than that of the OG/Cur system, highlighting a significant improvement in stability. When exposed to UV light for 100 min, the OG/Cur/Cu^2+^ system showed a 49.64% improvement compared to the OG/Cur system. This result indicates that the interface network formed in the OG/Cur/Cu^2+^ system can resist prolonged UV exposure (100 min) and performs significantly better than the OG/Cur system.

## 4. Conclusions

This study demonstrates that the synergistic coordination between octyl gallate (OG) and Cu^2+^ at the oil-water interface successfully constructs a viscoelastic interfacial film, which serves as a robust barrier for the efficient encapsulation and protection of curcumin. FT-IR spectroscopy confirmed that this interfacial network is formed through coordination bonds between Cu^2+^ and the phenolic hydroxyl groups of OG, as well as the keto/enol structure of curcumin. The emulsion system (formulated with 0.3 wt% OG and 100 μL Cu^2+^) maintained excellent stability over 7 days of storage across all tested temperatures, with a curcumin retention rate of 91.6% after 100 min of UV irradiation. Consequently, this metal-phenolic coordination strategy significantly enhances the stability and bioavailability of curcumin in complex environments, providing a novel and effective technical approach for its stable preservation and application.

The interfacial coordination strategy also offers a promising approach for stabilizing nutraceuticals in functional foods, such as beverages, fortified oils, and emulsion-based sauces. In order to better apply to food, safety should be considered. Octyl gallate is an approved food additive, and its amphiphilicity ensures its potential in multiphase systems. Copper is an essential trace element, but there are strict standards for its acceptable daily intake. To avoid potential safety issues, the concentrations of octyl gallate and copper ions used in our emulsion system are relatively low. Future research could explore the use of other food-grade essential mineral ions, such as zinc (Zn^2+^) or iron (Fe^3+^) ions, as alternatives to extend the generality of this strategy.

## Figures and Tables

**Table 1:** The diffusion coefficients (K_diff_) of the complexes under different Cu^2+^ concentrations at the oil-water interface.

Concentration (mM)	K_diff_ (mN/m/s^1/2^)
0.001	0.043 ± 0.007
0.003	0.052 ± 0.005
0.005	0.089 ± 0.004
0.007	0.142 ± 0.006

**Table 2:** The diffusion coefficients (K_diff_) of the complexes under different metal ions at the oil-water interface.

Metal Ions	K_diff_ (mN/m/s^1/2^)
Ca^2+^	0.014 ± 0.002
Mg^2+^	0.088 ± 0.004
Zn^2+^	0.035 ± 0.005
Cu^2+^	0.142 ± 0.006
Fe^2+^	0.123 ± 0.006

## Data Availability

The original contributions presented in the study are included in the article/Supplementary Materials, further inquiries can be directed to the corresponding authors.

## Supplementary Materials

The following supporting information can be downloaded at: https://www.mdpi.com/article/10.3390/foods15020265/s1, Figure S1: Optimization of the basic emulsion components; Figure S2: Backscattering and Turbiscan stability index of the four emulsions; Figure S3: Interfacial tension at emulsion with Cu^2+^ concentration of 0.008 mM. References [26,35,55,56,57,58,59,60,61] are cited in the supplementary materials.

## Abbreviations

The following abbreviations are used in this manuscript:

OG	Octyl gallate
Cur	Curcumin
BS	Backscattering
TSI	Turbiscan stability index

## References

[1] Xu X.-Y., Meng X., Li S., Gan R.-Y., Li Y., Li H.-B. (2018). Bioactivity, Health Benefits, and Related Molecular Mechanisms of Curcumin: Current Progress, Challenges, and Perspectives. Nutrients.

[2] Racz L.Z., Racz C.P., Pop L.-C., Tomoaia G., Mocanu A., Barbu I., Sárközi M., Roman I., Avram A., Tomoaia-Cotisel M. (2022). Strategies for Improving Bioavailability, Bioactivity, and Physical-Chemical Behavior of Curcumin. Molecules.

[3] Quichaba M.B., Moreira T.F.M., de Oliveira A., de Carvalho A.S., de Menezes J.L., Gonçalves O.H., de Abreu Filho B.A., Leimann F.V. (2023). Biopreservatives against foodborne bacteria: Combined effect of nisin and nanoncapsulated curcumin and co-encapsulation of nisin and curcumin. J. Food Sci. Technol..

[4] Araiza-Calahorra A., Akhtar M., Sarkar A. (2018). Recent advances in emulsion-based delivery approaches for curcumin: From encapsulation to bioaccessibility. Trends Food Sci. Technol..

[5] Jiang L., Xia N., Wang F., Xie C., Ye R., Tang H., Zhang H., Liu Y. (2022). Preparation and characterization of curcumin/β-cyclodextrin nanoparticles by nanoprecipitation to improve the stability and bioavailability of curcumin. LWT.

[6] Kharat M., Du Z., Zhang G., McClements D.J. (2017). Physical and Chemical Stability of Curcumin in Aqueous Solutions and Emulsions: Impact of pH, Temperature, and Molecular Environment. J. Agric. Food Chem..

[7] Misra S., Pandey P., Mishra H.N. (2021). Novel approaches for co-encapsulation of probiotic bacteria with bioactive compounds, their health benefits and functional food product development: A review. Trends Food Sci. Technol..

[8] Liu K., Chen Y.-Y., Pan L.-H., Li Q.-M., Luo J.-P., Zha X.-Q. (2022). Co-encapsulation systems for delivery of bioactive ingredients. Food Res. Int..

[9] Espitia P.J.P., Fuenmayor C.A., Otoni C.G. (2019). Nanoemulsions: Synthesis, Characterization, and Application in Bio-Based Active Food Packaging. Compr. Rev. Food Sci. Food Saf..

[10] Li Y., Miao Y., Yang L., Zhao Y., Wu K., Lu Z., Hu Z., Guo J. (2022). Recent Advances in the Development and Antimicrobial Applications of Metal—Phenolic Networks. Adv. Sci..

[11] Chen W., Ma S., Wang Q., McClements D.J., Liu X., Ngai T., Liu F. (2022). Fortification of edible films with bioactive agents: A review of their formation, properties, and application in food preservation. Crit. Rev. Food Sci. Nutr..

[12] Hu X., Ma T., Pan H., Chen S., Liu D. (2025). Direct assembly of tannic acid-based metal-phenolic networks at the oil-water Interface for emulsion stabilization. Food Res. Int..

[13] Khorasani M.Y., Langari H., Sany S.B.T., Rezayi M., Sahebkar A. (2019). The role of curcumin and its derivatives in sensory applications. Mater. Sci. Eng. C.

[14] Ferreira I., Costa M., Losada-Barreiro S., Paiva-Martins F., Bravo-Díaz C. (2018). Modulating the interfacial concentration of gallates to improve the oxidative stability of fish oil-in-water emulsions. Food Res. Int..

[15] Li W., Huang D., Jiang Y., Liu Y., Li F., Huang Q., Li D. (2021). Preparation of pickering emulsion stabilised by Zein/Grape seed proanthocyanidins binary composite. Int. J. Food Sci. Technol..

[16] Yang D., Wang L., Zhang L., Wang M., Li D., Liu N., Liu D., Zhao M., Yao X. (2024). Construction, characterization and bioactivity evaluation of curcumin nanocrystals with extremely high solubility and dispersion prepared by ultrasound-assisted method. Ultrason. Sonochem..

[17] Zhang X., Wei Z., Wang X., Wang Y., Tang Q., Huang Q., Xue C. (2022). Fabrication and characterization of core-shell gliadin/tremella polysaccharide nanoparticles for curcumin delivery: Encapsulation efficiency, physicochemical stability and bioaccessibility. Curr. Res. Food Sci..

[18] Wu Q., Hu X., Lu S., Xu B., Bai C., Ma T., Song Y. (2025). Study on the interactions and interfacial behaviors of cellulose nanocrystal/β-lactoglobulin complexes for pickering emulsions. Food Hydrocoll..

[19] Sun F., Li Z., Kong S., Ma X., Liu Y., Yang N. (2025). Linear and nonlinear interfacial rheology of responsive microgels at the oil-water interface. Food Hydrocoll..

[20] Dai C., Han S., Ma C., McClements D.J., Xu D., Chen S., Liu X., Liu F. (2024). High internal phase emulsions stabilized by pea protein isolate-EGCG-Fe^3+^ complexes: Encapsulation of β-carotene. Food Hydrocoll..

[21] McClements D.J., Gumus C.E. (2016). Natural emulsifiers—Biosurfactants, phospholipids, biopolymers, and colloidal particles: Molecular and physicochemical basis of functional performance. Adv. Coll. Interface Sci..

[22] Jafari S.M., Assadpoor E., He Y., Bhandari B. (2008). Re-coalescence of emulsion droplets during high-energy emulsification. Food Hydrocoll..

[23] Kong Y., Chen J., Hong Z., Huang Q. (2025). The role of yeast dietary fiber-derived proteins and polysaccharides in emulsion stabilization: Interfacial effects and stabilizing mechanisms. Food Chem..

[24] Wu D., Dai Y., Huang Y., Gao J., Liang H., Eid M., Deng Q., Zhou B. (2020). Metal–Phenolic Network Covering on Zein Nanoparticles as a Regulator on the Oil/Water Interface. J. Agric. Food Chem..

[25] Leiva-Vega J., Villalobos-Carvajal R., Ferrari G., Donsì F., Zúñiga R.N., Shene C., Beldarraín-Iznaga T. (2020). Influence of interfacial structure on physical stability and antioxidant activity of curcumin multilayer emulsions. Food Bioprod. Process..

[26] Diao X., Jia R., Wang Y., Liu G., Chen X., Liu D., Guan H. (2024). The physicochemical properties, microstructure, and stability of diacylglycerol-loaded multilayer emulsion based on protein and polysaccharides. LWT.

[27] Zhang Z., Liu Y., Gao Y., Huo J., Dong S., Liu L., Li S. (2025). Sustained-release effect of eggshell powder microcapsules on lavender essential oil. J. Food Eng..

[28] Cai X., Wang Y., Du X., Xing X., Zhu G. (2020). Stability of pH-responsive Pickering emulsion stabilized by carboxymethyl starch/xanthan gum combinations. Food Hydrocoll..

[29] Shao P., Qiu Q., Chen H., Zhu J., Sun P. (2017). Physicochemical stability of curcumin emulsions stabilized by Ulva fasciata polysaccharide under different metallic ions. Int. J. Biol. Macromol..

[30] Gao Y., Xu Z., Jin M., Wang X., Fan Z., Jiang L., Sui X. (2023). Ability of soy protein derived amyloid fibrils to stabilize aqueous two-phase system and effect of pH on the system. Food Hydrocoll..

[31] Zhou L., Xu G., Zhang Z., Li H., Yao P. (2018). Surface activity and safety of deamidated zein peptides. Coll. Surf. A Physicochem. Eng. Asp..

[32] Šimunková M., Biela M., Štekláč M., Hlinčík A., Klein E., Malček M. (2022). Cu(II) complexes of flavonoids in solution: Impact of the Cu(II) ion on the antioxidant and DNA-intercalating properties. J. Mol. Liq..

[33] Wu D., Zhou B., Wang S., Pei Y., Li B., Liang H. (2021). Pickering Emulsion Stabilized by Metal-Phenolic Architectures: A Straightforward In Situ Assembly Strategy. J. Agric. Food Chem..

[34] Zhen S., Zhang B., He S., Zhao C., Zhang Y., Ghorani B., Emadzadeh B., Yang N. (2026). Enhancement of the interfacial behaviors of β-lactoglobulin fibrils of different morphology by calcium ions. Food Hydrocoll..

[35] Marquez R., Ontiveros J.F., Nardello-Rataj V., Sanson N., Lequeux F., Molinier V. (2024). Formulating stable surrogate wood pyrolysis oil-in-oil (O/O) emulsions: The role of asphaltenes evidenced by interfacial dilational rheology. Chem. Eng. J..

[36] Bera A., Sarkar T., Upadhyay A., Hussain A. (2025). First-row transition metal complexes of naturally occurring anticancer chelators for cancer treatment. Coord. Chem. Rev..

[37] Chen Z., Świsłocka R., Choińska R., Marszałek K., Dąbrowska A., Lewandowski W., Lewandowska H. (2024). Exploring the Correlation Between the Molecular Structure and Biological Activities of Metal–Phenolic Compound Complexes: Research and Description of the Role of Metal Ions in Improving the Antioxidant Activities of Phenolic Compounds. Int. J. Mol. Sci..

[38] Geng H., Zhong Q.-Z., Li J., Lin Z., Cui J., Caruso F., Hao J. (2022). Metal Ion-Directed Functional Metal—Phenolic Materials. Chem. Rev..

[39] Tao Y., Cai J., Wang P., Chen J., Zhou L., Zhang W., Xu X. (2024). Application of rheology and interfacial rheology to investigate the emulsion stability of ultrasound-assisted cross-linked myofibrillar protein: Effects of oil phase types. Food Hydrocoll..

[40] Halevas E., Pekou A., Papi R., Mavroidi B., Hatzidimitriou A.G., Zahariou G., Litsardakis G., Sagnou M., Pelecanou M., Pantazaki A.A. (2020). Synthesis, physicochemical characterization and biological properties of two novel Cu(II) complexes based on natural products curcumin and quercetin. J. Inorg. Biochem..

[41] Chen Y., Tai K., Ma P., Su J., Dong W., Gao Y., Mao L., Liu J., Yuan F. (2021). Novel γ-cyclodextrin-metal–organic frameworks for encapsulation of curcumin with improved loading capacity, physicochemical stability and controlled release properties. Food Chem..

[42] Kalinowska M., Gryko K., Gołębiewska E., Świderski G., Lewandowska H., Pruszyński M., Zawadzka M., Kozłowski M., Sienkiewicz-Gromiuk J., Lewandowski W. (2022). Fe(III) and Cu(II) Complexes of Chlorogenic Acid: Spectroscopic, Thermal, Anti-/Pro-Oxidant, and Cytotoxic Studies. Materials.

[43] Massoni M., Clavijo J.C.T., Colina-Vegas L., Villarreal W., Dias J.S.M., da Silva G.A.F., Ionta M., Soares M., Ellena J., Dorigueto A.C. (2017). Propyl gallate metal complexes: Circular dichroism, BSA-binding, antioxidant and cytotoxic activity. Polyhedron.

[44] Shen X., Mo Y., Ren J., Liu K., Sun S., Deng C. (2025). Inhibition of xanthine oxidase by copper-gallate coordination polymers and its mechanistic study. J. Mol. Struct..

[45] Cai D., Wang X., Wang Q., Tong P., Niu W., Guo X., Yu J., Chen X., Liu X., Zhou D. (2024). Controlled release characteristics of alkyl gallates and gallic acid from β-cyclodextrin inclusion complexes of alkyl gallates. Food Chem..

[46] El-Megharbel S.M., Hamza R.Z. (2022). Synthesis, spectroscopic characterizations, conductometric titration and investigation of potent antioxidant activities of gallic acid complexes with Ca (II), Cu (II), Zn(III), Cr(III) and Se (IV) metal ions. J. Mol. Liq..

[47] Huang L., Zhao X., Zhang S., Xu Y., Wei H., Zhang M., Feng H., Wang X., Zheng Y., Zheng X. (2025). Metal polyphenol CA-V nanozymes promote acute wound healing by destroying the ROS-inflammation cascade cycle. Mater. Today Bio.

[48] Santoso S.P., Angkawijaya A.E., Cheng K.-C., Lin S.-P., Hsu H.-Y., Hsieh C.-W., Rahmawati A., Shimomura O., Ismadji S. (2025). Unlocking the Potential of Gallic Acid-Based Metal Phenolic Networks for Innovative Adsorbent Design. Molecules.

[49] Xue S., Tan W., Mao S., Pan H., Ye X., Donlao N., Tian J. (2025). Polyphenol-Based Functional Materials: Structural Insights, Composite Strategies, and Biomedical Applications. Adv. Sci..

[50] Kumar R., Uppal S., Kaur K., Mehta S.K. (2020). Curcumin nanoemulsion as a biocompatible medium to study the metal ion imbalance in a biological system. J. Mol. Liq..

[51] Wang D., Kim D., Shin C.-H., Zhao Y., Park J.-S., Ryu M. (2019). Evaluation of epigallocatechin gallate (EGCG) to remove Pb(II) using spectroscopic and quantum chemical calculation method. Environ. Earth Sci..

[52] Chen X., Li B., Ji S., Wu D., Cui B., Ren X., Zhou B., Li B., Liang H. (2023). Small molecules interfacial assembly regulate the crystallization transition process for nobiletin stabilization. Food Chem..

[53] Sun Y., Zhang S., Xie F., Zhong M., Jiang L., Qi B., Li Y. (2021). Effects of covalent modification with epigallocatechin-3-gallate on oleosin structure and ability to stabilize artificial oil body emulsions. Food Chem..

[54] Ejima H., Richardson J.J., Liang K., Best J.P., van Koeverden M.P., Such G.K., Cui J., Caruso F. (2013). One-Step Assembly of Coordination Complexes for Versatile Film and Particle Engineering. Science.

[55] Ho T.M., Razzaghi A., Ramachandran A., Mikkonen K.S. (2022). Emulsion characterization via microfluidic devices: A review on interfacial tension and stability to coalescence. Adv. Coll. Interface Sci..

[56] Pan Y., Liu L., Li J., Zhu B., Li X., Cheng J., Muneeb M., Kouame K.J.E., Jiang X. (2024). Enhancing the physical stability and bioaccessibility of curcumin emulsions through the interaction of whey protein isolate and soybean lecithin. Food Biosci..

[57] Chen Z., Zhao Z., Wang W., Ye Q., Xiao J. (2024). Simulating the behavior of antioxidant to explore the mechanisms of oxidative stability in Pickering emulsion. Food Chem..

[58] Zhao G., Luo Y., Li Q., Zhang M., Yin F., Zhou D. (2023). Exploring the antioxidant “cut-off effect” of gallic acid alkyl esters in dried oysters (*Crassostrea gigas*) during storage. Food Biosci..

[59] Yu Q., Wu H., Fan L. (2024). Formation of casein and maltodextrin conjugates using shear and their effect on the stability of total nutrient emulsion based on homogenization. Food Hydrocoll..

[60] Lin M., Chen Y., Shi L., Zhang Y., Liu S., Liu Z., Weng W., Ren Z. (2025). High internal-phase Pickering emulsions constructed using myofibrillar proteins from large yellow croaker: Effect of glycerol. Int. J. Biol. Macromol..

[61] Cai X., Du X., Zhu G., Shi X., Chen Q. (2023). Fabrication of carboxymethyl starch/xanthan gum combinations Pickering emulsion for protection and sustained release of pterostilbene. Int. J. Biol. Macromol..

