# Supplementary material for: Metal Ion-Mediated Interfacial Coordination Complexation in Octyl Gallate-Curcumin Emulsions: Enhanced Stability and Curcumin Protection
Source: Foods. 2026 Jan 11;15(2):265. doi: 10.3390/foods15020265 (PMC12840199; doi:10.3390/foods15020265)
Supplement: Supplementary file 1 [file foods-15-00265-s001.zip › foods-4052974-supplementary.pdf]

Supplementary Materials

1. Supplementary Method

1.1. Preparation method of basic emulsion

Curcumin was dissolved in corn oil (1 mg/mL) with heating at 60 °C under stirring to obtain complete dissolution. Various alkyl gallates (propyl gallate, octyl gallate, lauryl gallate, and stearyl gallate) were then added at different concentrations (0.8 wt, 1.0 wt, 1.5 wt, and 2.0 wt% based on oil phase) and stirred until fully dissolved to form the oil phase. The aqueous phase was buffer solution.

The oil phase was added dropwise to the aqueous phase at three oil-to-water ratios (1:9, 1.5:8.5, and 2:8, v/v). Emulsions were prepared using ultrasonic homogenization at 80 W with a pulse cycle (1s on, 5s off) for 3 min.

2. Supplementary Analysis

2.1. Optimization of the basic emulsion components

Figure S1A presents the macroscopic appearance of curcumin emulsions prepared with different alkyl gallates. In the propyl gallate group, all emulsions exhibited oil layer separation at the surface, likely due to excessive droplet coalescence in the system [1]. When distinct oil layer separation was observed, the emulsion system was considered unstable and thus excluded from subsequent particle size analysis.

For the octyl gallate-stabilized emulsions at 1:9 oil-to-water ratio, formulations with 1.0 wt%, 1.5 wt%, and 2.0 wt% emulsifier showed no visible oil separation. However, at 1.5:8.5 oil-to-water ratio, all concentrations except 2.0 wt% demonstrated oil layer formation. Complete phase separation occurred across all concentrations at 2:8 oil-to-water ratio.

The lauryl gallate group displayed similar separation patterns to octyl gallate but with thicker oil layers, indicating poorer emulsification performance. In the stearyl gallate group, relatively stable emulsions were only achieved at 1:9 oil-to-water ratio with 1.0 wt% or 1.5 wt% emulsifier, while all other parameter combinations resulted in oil layer separation.

Based on the these findings, octyl gallate, lauryl gallate and stearyl gallate were selected and used to prepare emulsions at 1.0, 1.5 and 2.0 wt% for an oil-to-water ratio of 1:9, and at 2.0 wt% for a oil-to-water ratio of 1.5:8.5. Particle size and polydispersity index (PDI) were subsequently determined for each formulation. As evidenced in Figure S1B,C, the octyl gallate-stabilized emulsions exhibited the smallest particle size and lowest PDI values, demonstrating its superior emulsification efficacy among the tested alkyl gallates [2]. The research results indicate that as the chain length increases, the emulsifying capacity of gallates first increased and then decreased, with octyl gallate demonstrating the best emulsification performance. This phenomenon may be attributed to the different alkyl chain lengths of gallates, which lead to variations in their hydrophilicity-lipophilicity properties, consequently affecting their distribution at the oil-water interface [3,4].

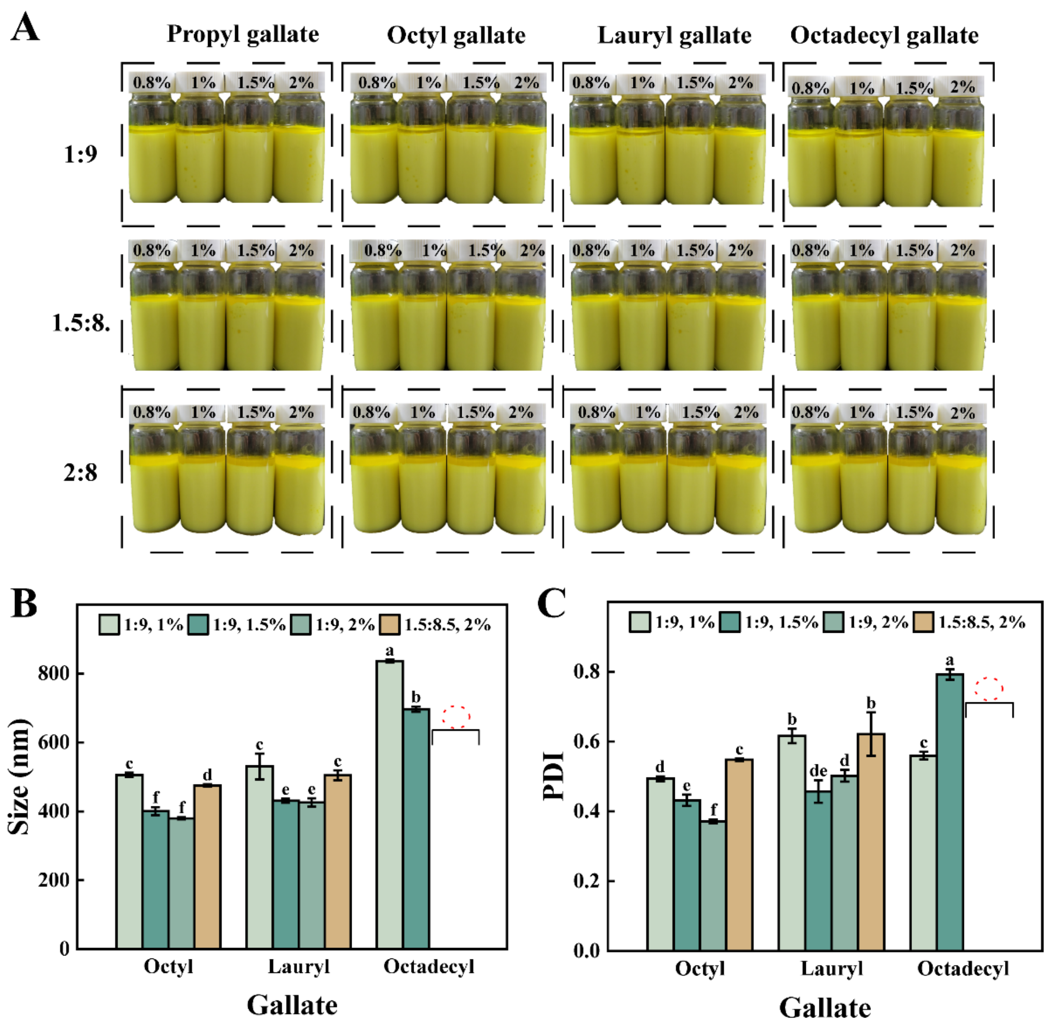

Figure S1. Macroscopic appearance (A) of emulsions with varying chain lengths, oil-to-water ratios, and emulsifier concentrations, particle size (B), polydispersity index (PDI) (C) of the selected emulsions.

The backscattering (BS) and Turbiscan stability index (TSI) of the four emulsions under different storage days were measured. The variation of BS can directly reflect the change of emulsion droplets over time. Greater variations in backscattered light indicates poorer stability of the emulsion [5]. Figure S2A-D presents the BS data obtained from continuous one-week monitoring of four different emulsions. The curves of emulsions with oil-to-water ratios of 1:9 and octyl gallate mass fractions of 1 wt% and 1.5 wt%, as well as 1.5:8.5 and 2%, exhibited a noticeable overall downward shift over time. This phenomenon may be attributed to the rupture of the interfacial film, dissolution of oil droplets, and subsequent occurrence of demulsification [6]. The emulsion with an oil-to-water ratio of 1.5:8.5 and OG mass fraction of 2% showed an increase in BS intensity at the top layer, which may indicate slight flocculation or coalescence of droplets [7]. In addition, the curves of the emulsion with an oil-water ratio of 1:9 and an OG mass fraction of 1% are generally superimposed closely with little fluctuation, indicating that the system is relatively stable overall. Furthermore, from the TSI shown in Figure S2E, it can be seen that the increase in TSI of this emulsion was the smallest, which also indicates that it is more stable compared to the other three emulsions [8]. In conclusion, based on the emulsion's particle size, homogeneity, fluorescence properties, and stability, the optimal

formulation was ultimately determined to be an oil-to-water ratio of 1:9 with 2% octyl gallate (OG) mass fraction.

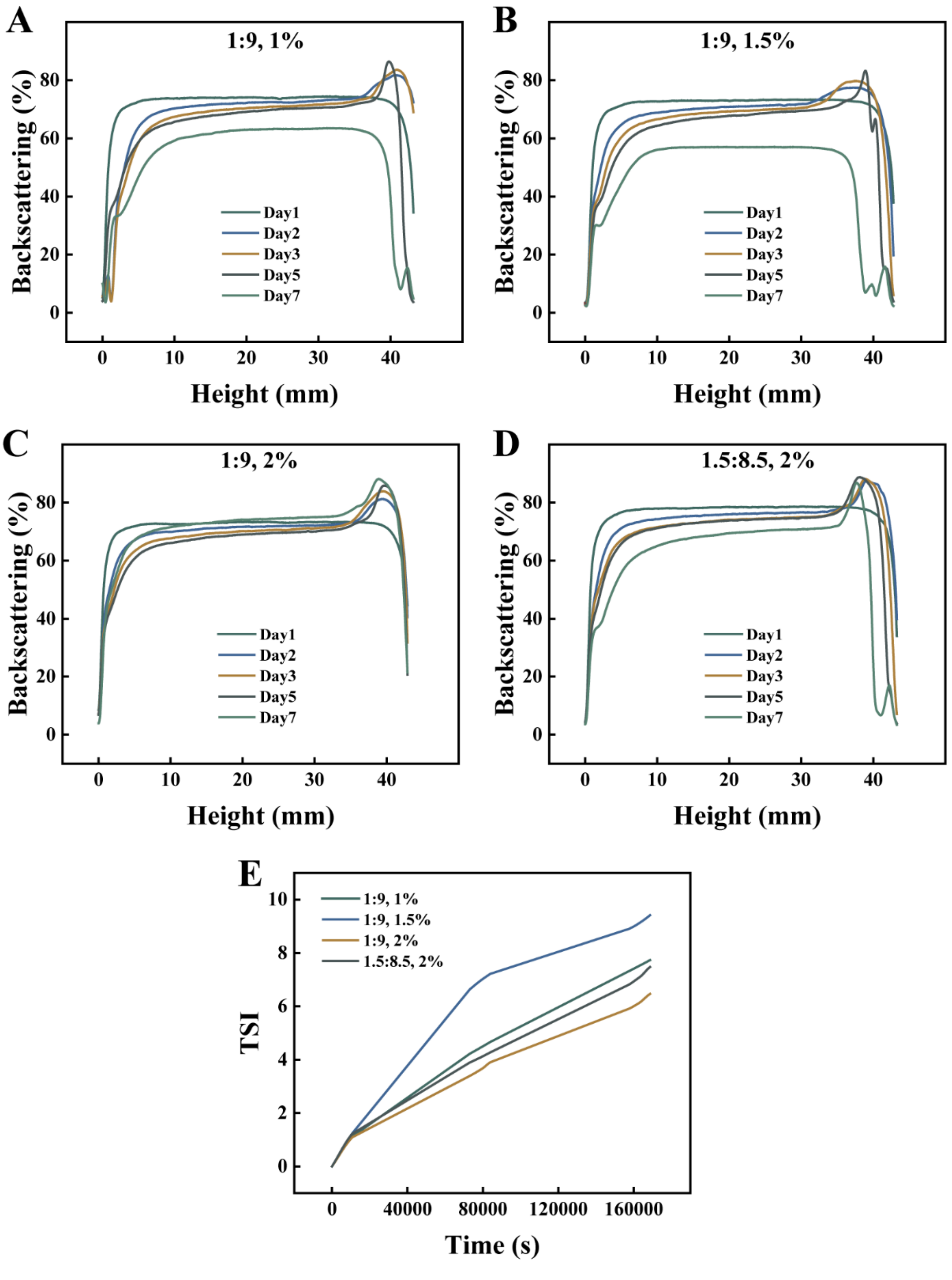

Figure S2. Backscattering (BS) (A-D) and Turbiscan stability index (TSI) (E) of the four emulsions.

2.2. The effect of Cu^{2+} concentration on interfacial tension

When 0.008 mM Cu^{2+} was added to the aqueous phase, the interfacial tension rapidly increased to 500 mN/m after 1750 s, followed by an abrupt decrease, as shown in Figure S3. This phenomenon primarily occurs because a solid-like film with high elastic modulus but very low viscous modulus forms at the interface. The droplets coated with this solid-like interfacial film no longer conform to the Laplace equation, thereby interfering with accurate interfacial tension measurements by the interfacial rheometer [9]. This further confirms the rapid cross-

linking of Cu^{2+}, octyl gallate and curcumin at the oil-water interface.

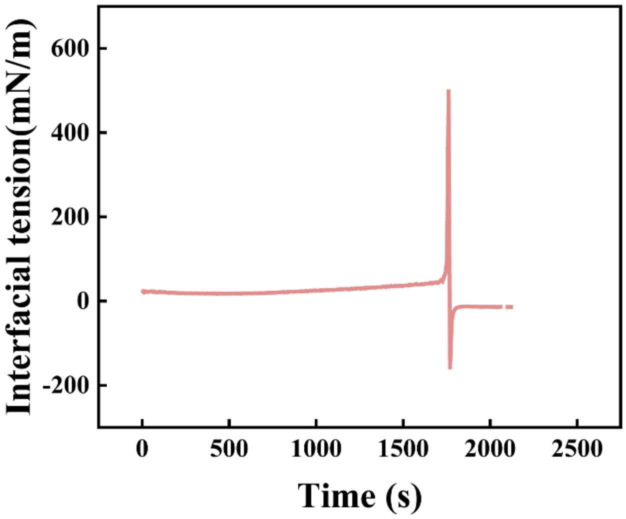

Figure S3. Interfacial tension at emulsion with Cu^{2+} concentration of 0.008 mM.

References:

1. Ho, T. M.; Razzaghi, A.; Ramachandran, A.; Mikkonen, K. S. Emulsion characterization via microfluidic devices: A review on interfacial tension and stability to coalescence. *Adv. Colloid Interface Sci.* **2022**, *299*, 102541.
2. Pan, Y.; Liu, L.; Li, J.; Zhu, B.; Li, X.; Cheng, J.; Muneeb, M.; Kouame, K. J. E.; Jiang, X. Enhancing the physical stability and bioaccessibility of curcumin emulsions through the interaction of whey protein isolate and soybean lecithin. *Food Biosci.* **2024**, *58*, 103676.
3. Chen, Z.; Zhao, Z.; Wang, W.; Ye, Q.; Xiao, J. Simulating the behavior of antioxidant to explore the mechanisms of oxidative stability in Pickering emulsion. *Food Chem.* **2024**, *447*, 138291.
4. Zhao, G.; Luo, Y.; Li, Q.; Zhang, M.; Yin, F.; Zhou, D. Exploring the antioxidant "cut-off effect" of gallic acid alkyl esters in dried oysters (*Crassostrea gigas*) during storage. *Food Biosci.* **2023**, *56*, 103197.
5. Yu, Q.; Wu, H.; Fan, L. Formation of casein and maltodextrin conjugates using shear and their effect on the stability of total nutrient emulsion based on homogenization. *Food Hydrocoll.* **2024**, *149*, 109533.
6. Diao, X.; Jia, R.; Wang, Y.; Liu, G.; Chen, X.; Liu, D.; Guan, H. The physicochemical properties, microstructure, and stability of diacylglycerol-loaded multilayer emulsion based on protein and polysaccharides. *LWT-Food sci technol.* **2024**, *196*, 115879.
7. Lin, M.; Chen, Y.; Shi, L.; Zhang, Y.; Liu, S.; Liu, Z.; Weng, W.; Ren, Z. High internal-phase Pickering emulsions constructed using myofibrillar proteins from large yellow croaker: Effect of glycerol. *Int. J. Biol. Macromol.* **2025**, *288*, 138605.
8. Cai, X.; Du, X.; Zhu, G.; Shi, X.; Chen, Q. Fabrication of carboxymethyl starch/xanthan gum combinations Pickering emulsion for protection and sustained release of pterostilbene. *Int. J. Biol. Macromol.* **2023**, *248*, 125963.
9. Marquez, R.; Ontiveros, J. F.; Nardello-Rataj, V.; Sanson, N.; Lequeux, F.; Molinier, V. Formulating stable surrogate wood pyrolysis oil-in-oil (O/O) emulsions: The role of asphaltenes evidenced by interfacial dilational rheology. *Chem. Eng. J.* **2024**, *495*, 153321.
